# Epidemiologic and phylogenetic analysis of the 2018 West Nile virus (WNV) outbreak in Israel demonstrates human infection of WNV lineage I

**DOI:** 10.2807/1560-7917.ES.2019.24.1.1800662

**Published:** 2019-01-03

**Authors:** Yaniv Lustig, Ruslan Gosinov, Neta Zuckerman, Yael Glazer, Laor Orshan, Danit Sofer, Eli Schwartz, Gili Schvartz, Yigal Farnoushi, Avishai Lublin, Oran Erster, Uri Shalom, Tamar Yeger, Orna Mor, Emilia Anis, Ella Mendelson

**Affiliations:** 1 Central Virology Laboratory, Ministry of Health, Tel-Hashomer, Israel; 2 Division of Epidemiology, Ministry of Health, Jerusalem, Israel; 3 Laboratory of Entomology, Ministry of Health, Jerusalem, Israel; 4 Institute of Tropical and Travel Medicine, Sheba Medical Center, Tel Hashomer, Ramat-Gan, Israel; 5 Kimron Veterinary Institute, Beit Dagan, Israel; 6 Ministry of Environmental Protection, Jerusalem, Israel; 7 Sackler Faculty of Medicine, Tel-Aviv University, Tel-Aviv, Israel; 8 Braun School of Public Health, Hebrew University and Hadassah, Israel

**Keywords:** West Nile virus, West Nile fever, WNV, West Nile Neuro-invasive Disease, Israel, WNV lineage I, mosquitoes, One Health

## Abstract

As at 12 November 2018, an outbreak of West Nile virus (WNV) was responsible for 139 WNV infection cases in Israel. Here, we characterise the epidemiology of the outbreak and demonstrate that only WNV lineage I was circulating in mosquitoes and responsible for WNV infection in humans. This suggests that the concurrence of the outbreak in Israel with WNV outbreaks in several European countries is not due to a common, more virulent WNV genotype.

Sequenced West Nile virus (WNV) strains in Israel typically belong to two distinct clusters within WNV lineage I [1,2] The concurrence of ongoing WNV outbreaks in Israel, as well as in several European countries, during the 2018 transmission season [4, 14] prompted us to investigate the epidemiological and phylogenetic characteristics of the outbreak in Israel and assess its relatedness to the outbreak in Europe.

## Epidemiological characteristics

WNV infection, a notifiable disease in Israel, is diagnosed by identification of immunoglobulin M (IgM) and G (IgG) antibodies in serum and CSF and WNV RNA in whole blood and urine samples [5].

**Figure 1:**
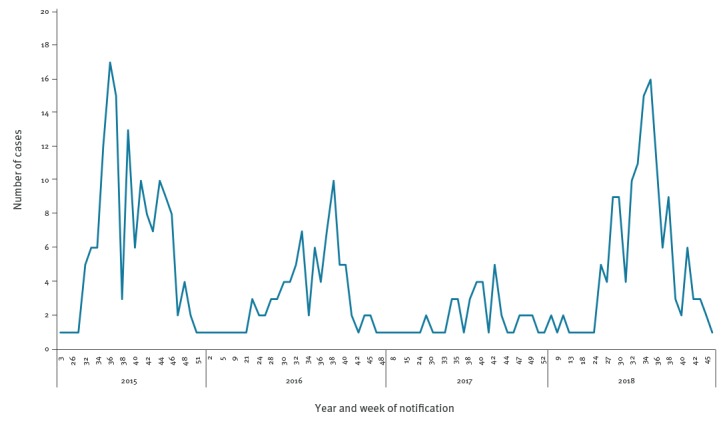
Number of West Nile fever cases by epidemiological week of diagnosis, Israel, 2015–18

As at 12 November 2018, 139 cases were diagnosed with WNV infection in Israel, of which 76 involved neurological complications and seven died. Sporadic cases were notified during weeks 9, 11, 15 and 17. A significant increase in the number of weekly cases has been observed since week 24 (Figure 1); the median age of cases was 63 years.

For comparison, in 2016 and 2017, 88 and 47 WNV cases were notified with median ages of 66 and 63 years, f:m ratio was 28:61 and 10:37, respectively. This was within the average number of cases of 79±44 for the years 2001 and 2017 in Israel [6,2]. In 2015, there was an outbreak of WNV with 149 notified cases. The 2015 outbreak started later in the WNV season (week 29) than the 2018 outbreak and the median age of cases was 68 years (f:m ratio 64:85).

In 2018, a One Health programme was initiated in Israel, which integrates WNV data obtained from humans, mosquitos, equine and birds. Within this initiative, the public health and veterinary services, Israel Nature and Park Authorities and the ministry of environmental protection report all WNV cases to the division of epidemiology, which, in turn, issues a monthly report during the WNV season [8].

**Figure 2:**
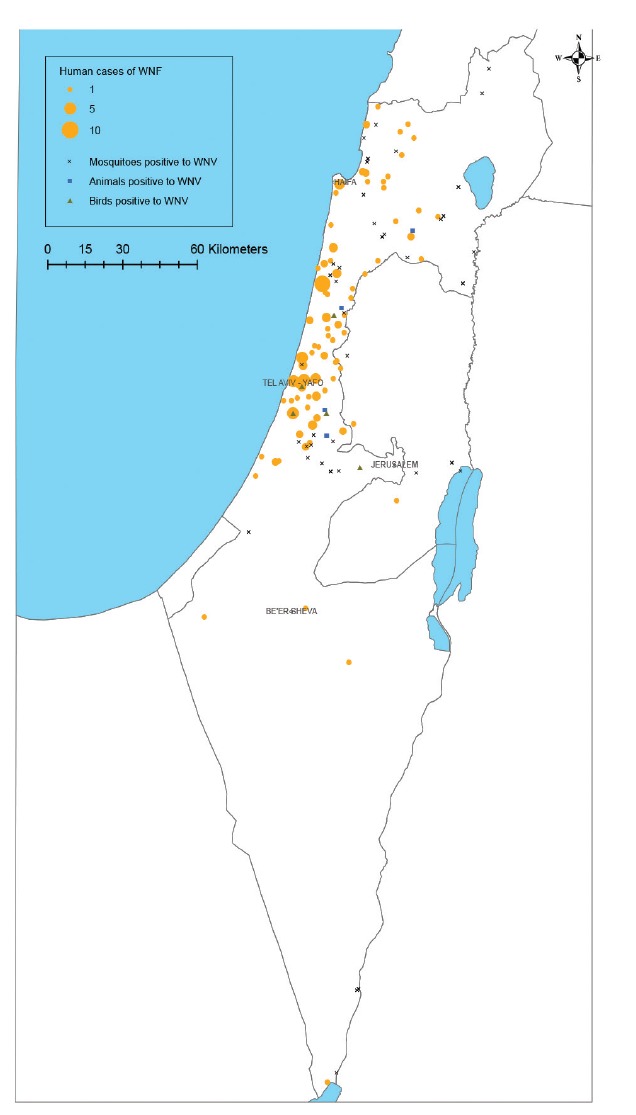
Spatial distribution of West Nile virus cases in humans, equine, birds and mosquitoes, Israel, 2018

WNF: West Nile fever; WNV: West Nile virus.

In Israel human WNV infections primarily occur in central Israel, which, in 2018, coincides with confirmed infection in birds, equine and mosquitoes (Figure 2).

Residence (by address) locations of acute WNV patients, trapping site locations of WNV-infected mosquito pools and locations of WNV-infected animals (equine and birds) detected in 2018 in Israel were overlaid on the geographical map of Israel.

## Phylogenetic investigation

**Figure 3:**
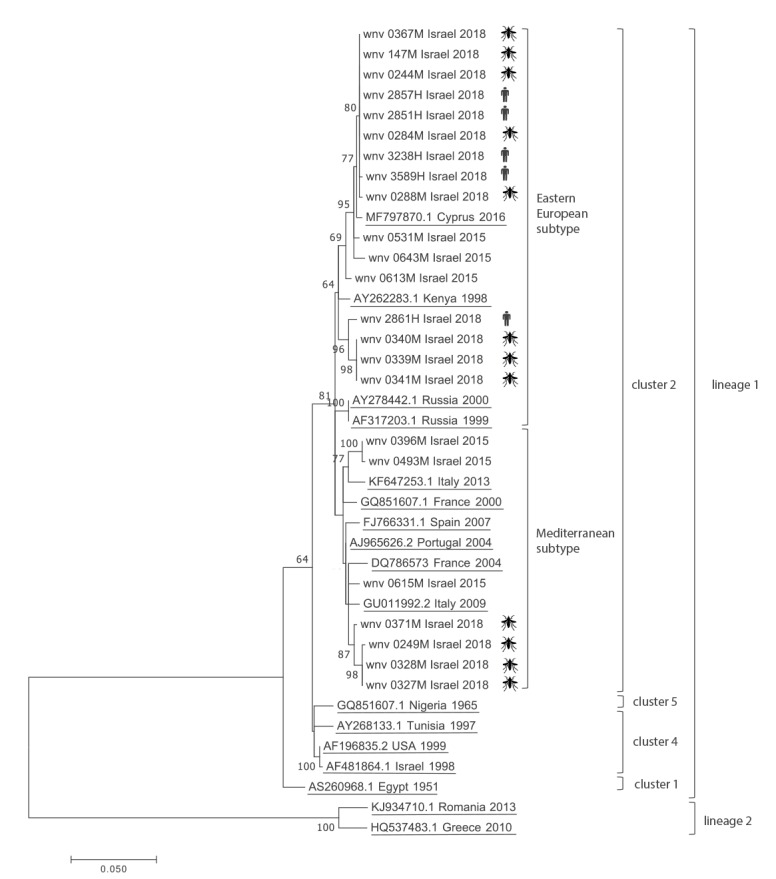
Phylogenetic tree of West Nile virus outbreak strains, Israel, 2015 and 2018

USA: United States of America; WNV: West Nile virus.

Seventeen qRT-PCR WNV-RNA-positive samples, obtained from WNV patients (five samples) and mosquito pools (12 samples) during 2018, were amplified by PCR for 890 nucleotides (nt) encoding part of the capsid and membrane (M) proteins and all of the premembrane (prM) protein [7]. Raw data was trimmed using Sequencher 5.4 (GeneCodes, Ann Arbor, Michigan, United States (US)) to generate 691 bp sequences of the 17 Israeli samples from 2018 which were aligned with six isolates from 2015 in Israel [2] and 16 WNV lineages I and II reference strains. The phylogenetic tree clearly demonstrates that all five samples obtained from humans clustered with the eastern European subtype of WNV lineage I, cluster 2 (Figure 3). Interestingly, the most closely related WNV reference strain was isolated from the first human case of WNV infection diagnosed in Cyprus in August 2016 [9,10]. WNV strains found in mosquitoes belonged to both the Mediterranean and eastern European subtypes of WNV lineage I, cluster 2.

Phylogenetic analysis was conducted on 40 WNV nt sequences of genes encoding the capsid, premembrane protein and membrane protein, with a total of 691 nt in the final alignment. Sequences included current Israeli WNV outbreak (Israel_2018), previous Israeli WNV outbreak (Israel_2015) and additional references. For sequences from the current 2018 outbreak, their origin is denoted by human/mosquito pictures. Trees were inferred by maximum likelihood based on the general time reversible model with invariant sites (GTR + I), predicted as the best-fit substitution model by JModelTest. The robustness of branching pattern was tested by 1,000 bootstrap replications and the percentage of successful bootstrap replicates is indicated at the nodes, where only values of > 60% are indicated.

## Discussion

In 2000, the largest West Nile fever (WNF) outbreak occurred in Israel with more than 400 WNF and West Nile Neuro-invasive Disease (WNND) cases [11-13]; since then, Israel has faced WNV outbreaks every few years [6]. While human WNV infections in Europe are prevalent [14], outbreaks are generally less frequent and occur every few years in distinct areas [15]. Notably, in 2018, a threefold increase in cases was recorded with simultaneous outbreaks in many countries across Europe, including countries with previously limited WNV circulation [14,15], and WNV lineage II was identified in Greece, Austria and Italy [4,16]. Similarly to the outbreak in Europe, the outbreak in Israel started early in the WNV season [14] but is likely unrelated to the European WNV outbreak, as only WNV lineage I was identified in all WNV human cases and WNV-positive mosquito pools.

As an endemic country, WNV morbidity in Israel is high in several areas and especially in the central part of the country [17]. Here, we show that outbreaks (including the 2018 outbreak) are not different in their geographical spread but rather in the intensity and attack rate of the disease [6]. On the other hand, WNV in Europe appears to have different dynamics with areas with high morbidity in 1 year may have no WNV cases in the following year [14,15]. These different patterns could be attributed to environmental conditions and/or seasonal WNV circulation. In this context, the current WNV season is unique due to the high morbidity and geographical spread of WNV in such an extensive area in central Europe and the Mediterranean basin.

Deciphering the underlying causes for WNV outbreaks is essential for prediction and prevention of future WNV infections. As several lineages, clusters and subtypes were identified circulating in Europe and the Mediterranean basin, analysis of their phylogenetic characteristics during an outbreak may determine the spread of the virus and potentially its origin. During the largest WNV outbreak in 2000 in Israel, at least two WNV clusters were infecting the human population [1], while in both 2015 and 2018, only one WNV type was responsible for all sequenced human infections [2]. Since 2010, most outbreaks in Europe were attributed to WNV lineage II [3] and two recent studies demonstrated that this WNV lineage II strain has significant genomic homogeneity in Europe, suggesting that it was introduced as a result of a locally-amplified single penetration event [18,19].

## Conclusion

The year 2018 has been exceptional in its severity with simultaneous and significant WNV outbreaks occurring across many European countries and in Israel. Moreover, infection in humans was detected very early in the transmission season raising the suspicion of emergence of a common, possibly more virulent, WNV strain. Our phylogenetic analysis demonstrates that all WNV sequences detected both in humans and in mosquitoes in Israel were related to WNV lineage I, unlike the outbreak in Europe, thus providing evidence for potential for different introductions of WNV lineages of the virus to Israel and Europe and implicating that indirect causes, e.g. climatic change, could be a potential cause of the simultaneous outbreak in Israel.
